# Structure of RiVax: a recombinant ricin vaccine

**DOI:** 10.1107/S0907444911026771

**Published:** 2011-08-09

**Authors:** Patricia M. Legler, Robert N. Brey, Joan E. Smallshaw, Ellen S. Vitetta, Charles B. Millard

**Affiliations:** a Naval Research Laboratories, 4555 Overlook Avenue, Washington, DC 20375, USA; b Soligenix Inc., 29 Emmons Drive, Suite C-10, Princeton, NJ 08540, USA; c Cancer Immunobiology Center, UT Southwestern Medical Center at Dallas, 6000 Harry Hines Boulevard, Dallas, TX 75390-8576, USA; d US Army Medical Research and Materiel Command, Frederick, MD 21702-5012, USA

**Keywords:** ricin, ribosome-inactivating proteins, protein engineering, immunogens, RiVax, B-cell epitopes

## Abstract

RiVax is a recombinant protein that is currently under clinical development as part of a human vaccine to protect against ricin poisoning. RiVax includes ricin A-chain (RTA) residues 1–267 with two intentional amino-acid substitutions, V76M and Y80A, aimed at reducing toxicity. Here, the crystal structure of RiVax was solved to 2.1 Å resolution and it was shown that it is superposable with that of the ricin toxin A-chain from *Ricinus communis* with a root-mean-square deviation of 0.6 Å over 258 C^α^ atoms. The RiVax structure is also compared with the recently determined structure of another potential ricin-vaccine immunogen, RTA 1–33/44–198 R48C/T77C. Finally, the locations and solvent-exposure of two toxin-neutralizing B-cell epitopes were examined and it was found that these epitopes are within or near regions predicted to be involved in catalysis. The results demonstrate the composition of the RiVax clinical material and will guide ongoing protein-engineering strategies to develop improved immunogens.

## Abstract 2

The X-ray crystal structure (at 2.1 Å resolution) of an immunogen under development as part of a ricin vaccine for humans is presented and structure-based analysis of the results was conducted with respect to related proteins and the known determinants for inducing or suppressing the protective immune response.

## 1. Introduction

Ricin is a heterodimeric ribosome-inactivating protein (RIP) that is available worldwide in ton quantities as a byproduct of castor-oil production from the plant *Ricinus communis*. The potent cytotoxicity of ricin may be beneficial for chemotherapy in humans, but has also been associated with misuse for political assassination, suicide and chemical warfare (Millard & LeClaire, 2007 ▶). The median lethal dose of ricin is in the range 1–10 µg kg^−1^ if administered to laboratory animals parenterally or by inhalation; recent work *in vitro* suggests that it may be possible to enhance the potency of ricin by genetic engineering (Olson *et al.*, 2004 ▶). A dose-dependent latent period of 4–­24 h after poisoning limits prompt diagnosis of ricin exposure, and irreversible toxin internalization by target cells renders post-exposure therapeutic intervention difficult. There is no approved antidote or treatment, but vaccination offers a practical prophylactic strategy to protect selected populations at risk of ricin exposure.

The crystal structure of the ricin A-chain (RTA; Rutenber *et al.*, 1991 ▶) has served as the starting point for several promising vaccine concepts (Compton *et al.*, 2011 ▶; Marsden *et al.*, 2005 ▶; Olson *et al.*, 2004 ▶; Vitetta *et al.*, 2006 ▶). In addition to vaccine development, there is interest in identifying structural correlates of ricin immunity to modulate the humoral immune response and extend the clinical use of RTA in ligand-targeted therapy (Chrunyk *et al.*, 1993 ▶; Sharp *et al.*, 2009 ▶; Smallshaw *et al.*, 2002 ▶).

RTA is an efficient N-glycosidase that catalyzes the hydrolysis of a specific adenine within the 28S rRNA of the 60S ribosomal subunit, leading to cell death. The hydrolysis reaction catalyzed by RTA is thought to proceed *via* a dissociative mechanism with an oxocarbenium transition state (Roday *et al.*, 2004 ▶). Glu177 in the active site stabilizes the developing positive charge on the substrate ribosyl ring, while Tyr80 and Tyr123 have been proposed to activate the leaving group by π-stacking with the adenine (Ghanem *et al.*, 2009 ▶). The enzymatic activity of RTA is the primary source of toxicity and therefore must be attenuated for safe vaccine development; this can be achieved by the introduction of a single point mutation, Y80A (Smallshaw *et al.*, 2002 ▶).

Based upon clinical observations and laboratory studies, it has been proposed that RTA harbors a second potential type of toxicity for humans that stems from a specific tripeptide sequence, Leu74-Asp75-Val76 (Smallshaw *et al.*, 2002 ▶); this peptide has been associated with pulmonary vascular leak syndrome (VLS) and has been referred to as the vascular leak-inducing peptide (VLP). While VLS is not observed in all laboratory animal models, the syndrome was dose-limiting in human chemotherapy trials and consequently is of concern for optimizing vaccine safety. Disruption of the VLP by a V76M mutation was shown to prevent RTA-induced weight loss, a biological marker of VLS, in specific animal models (Smallshaw *et al.*, 2002 ▶).

The RiVax immunogen is a double mutant of RTA that combines the Y80A mutation to inactivate catalysis with the V76M mutation to ensure the removal of any trace VLS activity from the immunogen. When properly stabilized and formulated with an adjuvant, RiVax elicited a protective humoral immune response in laboratory mice and safely induced ricin-protecting antibodies in human volunteers during Phase 1 clinical trials (Marconescu *et al.*, 2010 ▶; Smallshaw *et al.*, 2002 ▶; Vitetta *et al.*, 2006 ▶).

We designed and tested an alternative immunogen platform called RTA 1–33/44–198 (an RTA variant containing residues 1–198 with a deletion of loop residues 34–43), with the goal of limiting undesirable protein denaturation and aggregation by selective deletion of amino-acid residues from RTA that pack with the ricin B-chain (RTB) in the folded ricin heterodimer (McHugh *et al.*, 2004 ▶; Olson *et al.*, 2004 ▶). We have recently shown that RTA 1–33/44–198 can be stabilized further by the introduction of specific disulfide bonds (Compton *et al.*, 2011 ▶) and can fold into an immunoreactive form with numerous mutations in the VLP site (C. B. Millard, unpublished data). Both RiVax (Smallshaw *et al.*, 2005 ▶, 2007 ▶) and RTA 1–33/44–198 (Olson *et al.*, 2004 ▶) elicit neutralizing antibodies that protect animals against ricin exposure, but may differ in their complement of epitopes.

To characterize RiVax produced from a large-scale manufacturing run and compare it with RTA and related immunogens, we here present its X-ray crystal structure to 2.1 Å resolution (deposited as PDB entry 3srp). We discuss the effects of the Y80A/V76M mutations and the structural integrity of two neutralizing B-cell epitopes formed by residues Asn97–Phe108 or Leu161–Ile175, as well as an adjacent human T-cell epitope formed by residues Ile175–Glu185. We also compare RiVax with our recently determined X-ray structure of a synthetically stabilized RTA-based immunogen called RTA 1–33/44–198 R48C/T77C (PDB entry 3lc9). Comparison of the two immunogens with their parent molecule, RTA, permits us to assess the structural effects of the VLS substitutions, as well as the truncation of C-­terminal residues 199–267, on the secondary structure of the human T-cell epitope formed by residues Ile175–Glu185.

## 2. Materials and methods

RiVax was obtained from a 100 l *Escherichia coli* fermentation and purified essentially as described by Smallshaw and coworkers (Peek *et al.*, 2007 ▶; Smallshaw *et al.*, 2002 ▶). The protein was stored as a stock at 253 K in 10 m*M* histidine pH 6.0, 140 m*M* sodium chloride and 50% glycerol (a stabilizing excipient). The glycerol was removed by dialysis against 4 l 75 m*M* Tris–HCl pH 8.0, 1 m*M* EDTA at 277 K. Dithiothreitol was added to a final concentration of 5 m*M* using a 1.0 *M* stock. Crystals were grown by the hanging-drop method using a 1:1 ratio of protein (2.8 mg ml^−1^) to precipitant. The precipitant used was 30% ammonium sulfate containing 50 m*M* sodium acetate pH 4.2. Crystals appeared within 24 h. Crystals were soaked in Crystal Screen Cryo (Hampton Research, Aliso Viejo, California, USA) solution No. 20 [0.16 *M* ammonium sulfate, 0.08 *M* sodium acetate trihydrate pH 5.0, 20%(*w*/*v*) PEG 4000, 20%(*w*/*v*) glycerol] and flash-frozen in liquid nitrogen. Diffraction data were collected with a Bruker FR591 high-flux rotating-anode X-ray diffractometer (PROTEUM) and a SMART 6000 2K CCD detector. Initial phases were calculated using the structure of RTA. The structure was solved by molecular replacement using *AMoRe* (Navaza, 2001 ▶). Simulated annealing was carried out with *CNS* v.1.1 (Brünger *et al.*, 1998 ▶). The model was refined using *REFMAC*5 (Murshudov *et al.*, 2011 ▶) and model building and solvent addition was performed with *Coot* (Emsley & Cowtan, 2004 ▶).

## 3. Results and discussion

We ultimately seek to optimize the structure of RTA to arrive at cost-effective recombinant immunogens that are sufficiently stable for prolonged storage and field transport, fully safe for human use and can most efficiently elicit a high fraction of protective antibodies. Toward this goal, we compared the RiVax crystal structure with those of two closely related ricin vaccine immunogens: native RTA and a truncated RTA 1–33/44–198 derivative. The refinement statistics for RiVax are shown in Table 1 ▶. The X-ray crystal structure can be superposed onto that of RTA with a root-mean-square deviation (r.m.s.d.) of 0.6 Å over 258 Cα atoms, demonstrating that the Y80A and V76M mutations found in RiVax do not significantly perturb the overall protein fold or alter the conformation of residues corresponding to known neutralizing epitopes. Neither the active-site mutation (Y80A) nor the VLS mutation (V76M) significantly alters the side-chain conformations of adjacent residues.

The immunization of laboratory animals or humans with RTA or its derivatives generates a mixture of high-avidity antibodies, only some of which are capable of neutralizing the ricin toxin using *in vitro* or *ex vivo* assays (Lemley *et al.*, 1994 ▶; Maddaloni *et al.*, 2004 ▶; Mantis *et al.*, 2006 ▶; Neal *et al.*, 2010 ▶; O’Hara *et al.*, 2010 ▶). It is important to qualify our structural analysis with the point that RTA epitopes shown to be involved in toxin neutralization *in vitro* do not necessarily correlate with protection *in vivo* from ricin poisoning. We limit our discussion, therefore, to a few specific examples of ricin-neutralizing monoclonal antibodies (MAb) isolated from mouse or human sera for which the target epitopes have been characterized using peptide mapping or molecular modeling (Castelletti *et al.*, 2004 ▶; Lebeda & Olson, 1999 ▶; Lemley *et al.*, 1994 ▶; Neal *et al.*, 2010 ▶; Table 2 ▶).

**Figure 1:**
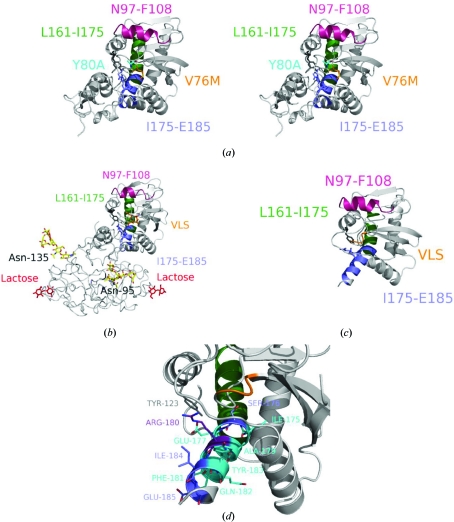
Comparison of the structures of RiVax, ricin holotoxin and the RTA 1–33/44–198 R48C/T77C immunogen. (*a*) Stereoview of the RiVax structure showing the two B-cell epitopes at Asn97–Phe108 and Leu161–Ile175 and the T-­cell epitope at Ile175–Glu185. The Asn97–Phe108 epitope bound by the UNIVAX 70/138 antibody is shown in magenta. The Leu161–Ile175 epitope bound by human neutralizing antibodies characterized by Castelletti *et al.* (2004 ▶) is shown in green. The Ile175–Glu185 T-cell epitope is shown in blue. The active-site residues within this epitope, Glu177 and Arg180, are shown as sticks; Tyr123, another active-site residue, is shown as dark gray sticks. The active-site mutation, Y80A, is shown in cyan. The mutation to the VLS site, V76M, is shown in orange. (*b*) Structure of the ricin AB toxin determined by Rutenber *et al.* (1991 ▶) (PDB entry 2aai). The A-chain is depicted by ribbons and the B-chain by tubes. Lactose (colored red) is reversibly bound to the carbohydrate-binding site. The N-linked sugars resolved at the glycosylation sites Asn95 and Asn135 are shown as yellow sticks. The putative immunological epitopes are colored as in (*a*). (*c*) Structure of the RTA 1–33/44–198 R48C/T77C disulfide-bonded variant based upon PDB entry 3lc9 (Compton *et al.*, 2011 ▶). The epitopes and VLS site are colored as in (*a*). The helix–turn–helix motif between residues Ile175 and Glu185 (blue) is found to be fully helical in the 1–33/44–198 R48C/T77C variant. (*d*) Mapping of residues which are believed to be important in T-­cell activation (Castelletti & Colombatti, 2005 ▶; shown in cyan) onto the helical segment between Ile175 and Glu185. Residues which do not affect T-cell activation when mutated to alanine are shown in blue. One residue, Arg180, had an intermediate affect on T-cell activation (purple). The figures were produced using *PyMOL* (DeLano, 2002 ▶).

One of the most potent neutralizing antibodies produced against RTA, called UNIVAX 70/138 (or R70), recognizes a solvent-exposed loop–helix–loop segment anchored by two β-strands between residues Asn97 and Phe108 (Lemley *et al.*, 1994 ▶; Fig. 1 ▶). The protective effects of some toxin-neutralizing antibodies can be explained by the functional location of their epitopes; for example, protective antibodies produced against the cell-binding domain of *Diphtheria* toxin may neutralize the toxin by physically preventing the B-chain from binding to the target cell surface (Pappenheimer *et al.*, 1972 ▶). RTB is known to contain a binding domain that binds to cell surface glycoproteins or glycolipids containing galactose, thereby facilitating toxin uptake (Fig. 1 ▶ *b*, based on PDB entry 2aai; Rutenber *et al.*, 1991 ▶; Ganguly & Mukhopadhyay, 2006 ▶). An examination of the 2aai crystal structure shows that the RTA epitope recognized by UNIVAX 70/138 is distant from RTB and its carbohydrate-binding sites, suggesting that this MAb does not neutralize ricin toxin by a direct steric effect on the lectin function of the toxin. The conformation of the structural region comprising RTA 99–106 has been discussed previously as an important epitope for generation of toxin-neutralizing antibodies (Lebeda & Olson, 1999 ▶) and substitutions that perturb this helix have been reported to modulate the catalytic activity of RTA directly in the absence of RTB (Olson *et al.*, 2004 ▶).

**Figure 2:**
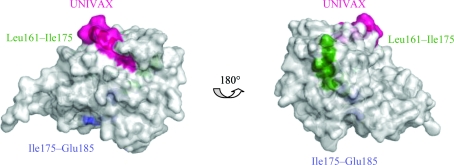
Surface representation of RiVax showing the exposure of each epitope. Colors for each epitope are the same as in Fig. 1 ▶(*a*).

Another murine MAb called GD-12 has been characterized that can neutralize toxin *in vitro* and protect mice against intraperitoneal or intragastric ricin exposure (Neal *et al.*, 2010 ▶). The GD-12 binding site is located near RTA residues Thr163–Met174 (TLA­RSFIICIQM) and overlaps with a human neutralizing B-cell epitope between residues Leu161 and Ile175 (Castelletti *et al.*, 2004 ▶). Examination of the GD-12 epitope in the X-­ray crystal structures suggests that unlike UNIVAX 70/138, the binding site for GD-12 is partially buried in the folded protein (Fig. 2 ▶).

The human B-cell neutralizing epitope identified at Leu161–Ile175 localizes to a helix–turn–helix (HTH) motif formed by helices 5 and 6 in RTA (Fig. 1 ▶). The HTH motif also encompasses an immunodominant human T-cell epitope, Ile175–Glu185, recognized by isolated T-cell clones (Castelletti & Colombatti, 2005 ▶). The HTH contains a type I reverse turn between Phe181 and Ile184 which connects the two antiparallel helices of RiVax. In contrast to RiVax or RTA, the crystal structure of the RTA 1–33/44–198 R48C/T77C immunogen reveals that truncation of the C-terminus converts the HTH into a helical segment. The segment between Ile175 and Glu185 has not previously been shown to be able to form a helix; its secondary structure appears to be dependent upon other motifs in the C-­terminal 199–267 residues.

The secondary structure of the Ile175–Glu185 segment may be an important structural determinant for peptide presentation on MHC molecules (Castelletti & Colombatti, 2005 ▶). The identification of this T-cell epitope has facilitated a strategy to circumvent an immune response to RTA-based chemotherapeutics by using altered peptide ligands which can modulate T-cell receptor affinities and subsequent T-cell activation (Adorini, 1993 ▶; Castelletti & Colombatti, 2005 ▶). In Fig. 1(*d*), the cyan-colored residues correspond to those which significantly reduce T-cell activation and are likely to bind to the T-­cell receptor. These residues localize to one face of the helix found in the 1–33/44–198 R48C/T77C structure and include the hydrophobic residues Ile175, Ala179, Phe181 and Tyr183. Castelletti and coworkers reported smaller effects on T-cell activation for the S176A, R180A, I184A and E185A variants. These mostly hydrophilic residues localize to the opposite face of the helix and may bind the MHC molecule (Kurata & Berzofsky, 1990 ▶; Sette *et al.*, 1989 ▶). While this segment of the protein has not previously been shown to be helical in RiVax or RTA, the helix observed in the RTA 1–33/44–198 variant may help to identify additional mutations to create altered peptide ligands.

Finally, we observe that essentially all biologically relevant B-cell and T-cell epitopes identified in RTA to date coincide with regions of the enzyme containing residues involved in substrate binding or catalysis. The active-site residue Glu177 localizes to a characterized T-cell epitope important in T-cell activation (Castelletti & Colombatti, 2005 ▶). Asp96 also interacts with a portion of the substrate (PDB entry 3hio; Hazes & Dijkstra, 1988 ▶). In the current model of sub­cellular trafficking of ricin, the toxin is endocytosed and the protein is transported to the Golgi and then to the endoplasmic reticulum (ER; Wesche, 2002 ▶). RTA is released from RTB, perhaps by reduction of a linking disulfide. RTA then unfolds and dislocates into the cytoplasm (Clarke & Fersht, 1993 ▶). The ribosome substrate of RTA resides within the target cell cytoplasm, to which antibodies would not easily have access. Binding of toxin-neutralizing antibodies is most likely to interfere with the steps preceding or following endocytosis; for example, RTA may need to undergo a particular conformational change during toxin binding to the target cell surface or to expose a surface which interacts with proteins that protect against lysosomal degradation (Moisenovich *et al.*, 2004 ▶); alternatively, antibodies might alter binding with a functional post-translational modification (*e.g.* an ADP-ribosylated amino acid; Glowacki *et al.*, 2002 ▶). Future structure-based studies of ricin-neutralizing antibodies are required to carefully test whether and how binding or occluding the RTA active site may perturb the complex process of toxin internalization and function.

## Supplementary Material

PDB reference: RiVax, 3srp

## Figures and Tables

**Table 1:** X-ray crystallography data-collection and refinement statistics Values in parentheses are for the outermost data shell.

Space group	*P*4_1_2_1_2
Unit-cell parameters (Å)	*a* = *b* = 66.56, *c* = 136.72
Wavelength (Å)	1.54
Resolution range (Å)	68.36–2.14 (2.23–2.14)
Unique reflections	17463 (1763)
*R*_merge_†	0.0988 (0.3993)
〈*I*/σ(*I*)〉	21.1 (4.4)
Completeness (%)	98.5 (87.4)
Multiplicity	12.4 (6.1)
Refinement statistics	
Resolution (Å)	59.9–2.1
No. of reflections	16508
*R* factor‡	0.217
*R*_free_§	0.254
No. of atoms	
Protein	2064
Solvent	151
Other	10
Average *B* factors (Å^2^)	
Protein	16.3
Solvent	21.6
R.m.s.d.s from ideal geometry	
Bond lengths (Å)	0.008
Bond angles (°)	0.980
Ramachandran plot	
Most favored regions (%)	92.6
Additional allowed regions (%)	7.4
Generously allowed regions (%)	0.0
Disallowed regions (%)	0.0

† *R*_merge_ = 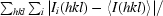 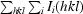.

‡ *R* factor = 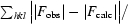 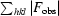.

§ *R*_free_ was calculated for a test set consisting of 5% of the total reflections.

**Table 2:** Regions of RTA structure potentially involved in MAb binding

RTA residues	Active-site residues or residues involved in substrate binding	Predicted unfolding regions†	B- or T-cell epitope(s)	Neutralizing antibody	Residues important in T-cell activation‡
1–117	Tyr80, Asp96	34–46	—	—	—
		80–100	Tyr91–Thr116 (Asn97–Phe108)	Mouse IgG1 (UNIVAX 70/138)	—
118–210	Tyr123, Glu177, Arg180	130-160	Leu161–Ile175	Human IgG	—
			Thr163–Met174	Mouse IgG1 (GD-12§)	—
			Ile175–Glu185	—	Ile175, Glu177, Phe181, Gln182, Tyr183, Glu185
211–276	—	—	—	—	—

† RTA regions proposed to be involved in the early stages of unfolding for residues 1–198 are based on previously published studies using molecular-dynamics and coarse-grain simulations (Compton *et al.*, 2011 ▶).

‡ Residues identified by Castelletti & Colombatti (2005 ▶).

§ Monoclonal antibody described by Roday *et al.* (2004 ▶).
